# Changes in neonatal inpatient resource utilization and cost drivers under payment reform: a five-year retrospective real-world data study

**DOI:** 10.3389/fped.2026.1943786

**Published:** 2026-09-21

**Authors:** Jinhui Hu, Juan Liu, Jing Qin, Zhaojun Pan

**Affiliations:** 1 Neonatal Medical Center, Affiliated Hospital of Yang Zhou University Medical College Huai'an maternal and Child health care Center, Huai'an, China; 2 Department of Finance, Affiliated Hospital of Yang Zhou University Medical College Huai'an maternal and Child health care Center, Huai'an, China

**Keywords:** diagnosis-intervention packet payment reform, diagnosis-Related groups, extremely preterm infant, hospitalization cost, length of stay, neonate

## Abstract

**Objective:**

To characterize temporal changes in neonatal inpatient case mix, resource utilization, and hospitalization costs from 2020 to 2024, with particular attention to cost-structure changes around the January 2022 payment reform, and to identify key clinical factors associated with hospitalization costs.

**Methods:**

This single-center retrospective study enrolled neonates admitted between January 2020 and December 2024 with valid total hospitalization costs and length of stay (LOS). Descriptive statistics characterized annual case mix and cost trends. Gestational age (GA)-standardized cost indices and daily cost intensity were calculated to assess resource consumption across different case mixes. With January 2022 designated as the reform index point, interrupted time series (ITS) analysis was applied to evaluate temporal changes in the proportions of procedural fees and Western medicine fees. Gamma generalized linear models (GLM), quantile regression, and ordinary least squares (OLS) regression were performed to examine factors associated with hospitalization costs.

**Results:**

A total of 12,011 neonates were included. Annual admissions declined from 2,986 in 2020 to 1,952 in 2024 (a 34.6% decrease), while the proportion of extremely preterm infants rose from 0.7% to 2.4% and the proportion requiring mechanical ventilation increased from 27.1% to 39.3%. The overall median annual hospitalization cost remained relatively stable (9,588 to 10,498 CNY), but the GA-standardized cost index consistently exceeded the unadjusted level (12,811 to 15,136 CNY). After the January 2022 reform index point, the proportion of procedural fees increased immediately by 32.30 percentage points (*P* < 0.001), while no concurrent significant change was observed in the proportion of Western medicine fees. Costs were highly concentrated, with the top 5% of patients across all study years accounting for 31.4% of total costs. Daily cost intensity was highest in extremely preterm infants (1,997 CNY/d). Multivariable analysis showed that LOS (exp β = 1.045) and mechanical ventilation (exp β = 1.81) were the most important factors associated with increased hospitalization costs.

**Conclusions:**

Neonatal admissions declined over the study period, while proportions of high-risk cases and cost concentration increased. LOS and mechanical ventilation were strongly associated with higher hospitalization costs, indicating that risk adjustment is essential in neonatal payment policies and resource allocation.

## 1 Introduction

With advances in perinatal medicine and neonatal intensive care technology, survival rates among high-risk neonates, including preterm infants and those with low birth weight, have improved markedly; however, the incidence of adverse outcomes has correspondingly increased (1, 2). Furthermore, prolonged hospitalization and intensive treatment place a substantial economic burden on both families and the healthcare system (3). In recent years, neonatal healthcare in China has been shaped by concurrent demographic changes and health insurance payment reforms. On one hand, the birth rate has continued to decline, and the population entered a phase of negative growth in 2022; on the other hand, improved neonatal rescue capabilities have enabled more extremely preterm infants to receive active treatment (1, 4). Simultaneously, the progressive implementation of diagnosis-related groups (DRG) and diagnosis-intervention packet (DIP) payment reforms, along with the national volume-based procurement (NVBP) program, may reshape cost structures through changes in payment constraints, pricing of medical services, and drug prices (5–7).

Prior studies have demonstrated that DRG/DIP reforms can affect hospitalization costs, LOS, and cost item composition, primarily manifested as reductions in pharmaceutical and consumable expenditures and increases in diagnostic and procedural service fees (8, 9). However, the existing evidence is largely derived from adult surgical, oncological, and cardiovascular populations. Research on neonatal hospitalization costs has largely focused on specific conditions, such as necrotizing enterocolitis, or selected preterm subgroups, including very preterm and extremely preterm infants, with relatively short observation periods and limited evaluation of case mix shifts, cost distribution heterogeneity, and pre- versus post-reform cost structural changes (10–12).

Therefore, using data from a tertiary maternal and child health hospital in eastern China covering 2020 to 2024, this study aims to analyze annual changes in admissions, case mix, hospitalization costs, and cost concentration; to apply daily cost intensity to characterize per-unit-time resource consumption by GA group; to use interrupted time series (ITS) analysis with January 2022 as the pre-specified index point to assess changes in cost composition; and to apply quantile regression and Gamma GLM with a log link function to examine key factors associated with costs at different expenditure levels. The findings are intended to inform DRG weight calibration, neonatal intensive care unit (NICU) resource allocation, and the management of neonatal hospitalization costs.

## 2 Methods

### 2.1 Study design

This study was conducted at the Affiliated Hospital of Yangzhou University Medical College Huai'an Maternal and Child Health Care Center, a tertiary Grade A specialized maternal and child hospital in eastern China with a neonatal department, a NICU, and well-established medical records and cost management systems. The study population comprised neonates discharged from the neonatal department between January 2020 and December 2024. Inclusion criteria were neonates with an age at admission of no more than 28 days. Exclusion criteria were: major congenital malformations or inborn errors of metabolism; incomplete key medical record information; hospitalization duration less than 24 h; and invalid records for total hospitalization cost or LOS. Of 12,036 original records, 25 records with total hospitalization costs or LOS of zero or less were excluded, leaving 12,011 patients for the annual trend, GA-stratified, and cost concentration analyses.

This study was conducted in accordance with the Declaration of Helsinki and was approved by the Ethics Committee of the Affiliated Hospital of Yangzhou University Medical College Huai'an Maternal and Child Health Care Center (approval number: 2025022). Informed consent was waived by the Ethics Committee because this was a retrospective study using anonymized data posing no additional risks to participants.

### 2.2 Data collection

A dedicated investigative team comprising two neonatologists with more than five years of clinical experience, one finance officer, and two certified medical record administrators was established. Patient data were collected from the hospital information system (HIS), electronic medical records, and paper-based medical records, including demographic and clinical characteristics, hospitalization information, primary diagnoses, and hospitalization costs. Variables collected included sex, GA at birth, birth weight (BW), LOS, NICU admission, and mechanical ventilation use. Patients were classified by GA into four groups: extremely preterm (< 28 weeks), very preterm (28 to 31 + 6 weeks), moderate-to-late preterm (32 to 36 + 6 weeks), and term (≥37 weeks). Mechanical ventilation was determined based on records of invasive or non-invasive ventilatory support, in conjunction with respiratory support treatment information.

Primary diagnoses included neonatal asphyxia, hypoxic-ischemic encephalopathy (HIE), intracranial hemorrhage, hyperbilirubinemia, transient tachypnea of the newborn (TTN), neonatal respiratory distress syndrome (NRDS), meconium aspiration syndrome (MAS), pneumothorax, pulmonary hemorrhage, pneumonia, sepsis, gastrointestinal hemorrhage, necrotizing enterocolitis (NEC), urinary tract infection, hypoglycemia, and anemia (13).

Total hospitalization cost was defined as the sum of all items recorded in the hospital billing system, including medical and nursing service fees, procedural service fees, laboratory diagnostic fees, imaging diagnostic fees, clinical diagnostic fees, pharmaceutical fees, antibacterial drug fees, disposable medical supply fees, and other fees. Two researchers independently entered data into Microsoft Excel 2021, and a third researcher verified the entries; any discrepancies were resolved by re-consulting the original medical records.

### 2.3 Derived indicators and statistical models

Detailed definitions of derived indicators and statistical models are provided in Supplementary Material S1**.** These include: (1) GA-standardized cost index and daily cost intensity (14); (2) ITS analysis (15); (3) cost concentration (16); and (4) regression models.

### 2.4 Statistical analysis

Statistical analyses were performed using Python 3.11, and GraphPad Prism 9.0 was used for figure preparation. Normally distributed data are presented as mean ± standard deviation; non-normally distributed data are presented as median (interquartile range, IQR). Categorical variables are presented as counts and percentages. Comparisons of non-normally distributed continuous variables between two groups used the Mann–Whitney *U* test, and comparisons among multiple groups used the Kruskal–Wallis *H* test. Annual monotonic trends were assessed using the Mann-Kendall test, and correlations between continuous variables were evaluated by Spearman rank correlation. ITS was implemented using segmented regression with Newey-West robust standard errors to account for potential autocorrelation and heteroscedasticity. Multivariable models were pre-specified to include variables with established clinical significance: sex, GA, BW, NICU admission, mechanical ventilation, and LOS; single-factor *P* values were not used as the sole criterion for variable selection. Multicollinearity was assessed using the variance inflation factor (VIF); a VIF below 5 was considered indicative of no substantial multicollinearity. Gamma GLM (with a log link function) served as the primary cost model; quantile regression was used to evaluate the heterogeneity of effects across different cost percentiles; and OLS served as a supplementary analysis. All tests were two-tailed, and statistical significance was set at *P* < 0.05.

## 3 Results

### 3.1 Annual admissions, case mix, and costs

A total of 12,011 neonates were included in the annual trend analysis, with 2,986 cases in 2020; 2,742 cases in 2021; 2,362 cases in 2022; 1,969 cases in 2023; and 1,952 cases in 2024. Admissions in 2024 were 34.6% lower than in 2020. Over the same period, the proportion of extremely preterm infants rose from 0.7% to 2.4%, very preterm infants from 4.7% to 6.3%, and moderate-to-late preterm infants from 24.2% to 26.6%, while the proportion of term infants declined from 69.9% to 64.2% (Table 1; Figure 1). The overall proportion of male infants was 56.6%.

**Table 1:** Annual clinical characteristics and case mix trends in neonates.

Characteristic	2020 (*n* = 2,986)	2021 (*n* = 2,742)	2022 (*n* = 2,362)	2023 (*n* = 1,969)	2024 (*n* = 1,952)	Overall (*n* = 12,011)
Admissions, *n*	2,986	2,742	2,362	1,969	1,952	12,011
Change from previous year, %	Reference	−8.2	−13.9	−16.6	−0.9	—
Extremely preterm (<28 wk), *n* (%)	21 (0.7)	23 (0.8)	44 (1.9)	24 (1.2)	47 (2.4)	159 (1.3)
Very preterm (28–31 + 6 wk), *n* (%)	139 (4.7)	108 (3.9)	125 (5.3)	76 (3.9)	122 (6.3)	570 (4.7)
Moderate-to-late preterm (32–36 + 6 wk), *n* (%)	724 (24.2)	668 (24.4)	584 (24.7)	499 (25.3)	519 (26.6)	2,994 (24.9)
Term (≥37 wk), *n* (%)	2,086 (69.9)	1,904 (69.4)	1,602 (67.8)	1,363 (69.2)	1,254 (64.2)	8,209 (68.3)
Missing GA, *n* (%)	16 (0.5)	39 (1.4)	7 (0.3)	7 (0.4)	10 (0.5)	79 (0.7)
Male, *n* (%)	1,718 (57.5)	1,527 (55.7)	1,371 (58.0)	1,071 (54.4)	1,117 (57.2)	6,804 (56.6)
NICU admission, *n* (%)	215 (7.2)	966 (35.2)	2,004 (84.8)	1,398 (71.0)	1,465 (75.1)	6,048 (50.4)
Mechanical ventilation, *n* (%)	808 (27.1)	865 (31.5)	824 (34.9)	711 (36.1)	768 (39.3)	3,976 (33.1)
In-hospital mortality, *n* (%)	12 (0.4)	9 (0.3)	9 (0.4)	4 (0.2)	7 (0.4)	41 (0.3)
Median total cost, CNY	9,841	10,498	10,415	9,588	9,775	10,055
GA-standardized cost index, CNY	12,811	15,136	14,749	14,020	13,734	—

GA, gestational age; NICU, neonatal intensive care unit. GA-standardized cost index: costs for each annual GA subgroup were weighted using the 2020 GA distribution as the reference, for descriptive comparison across years only; this index does not represent a fully case-mix-adjusted estimate or an overall median cost. GA subgroup proportions were calculated using validated gestational age data; 79 cases with missing GA are listed separately.

**Figure 1:**
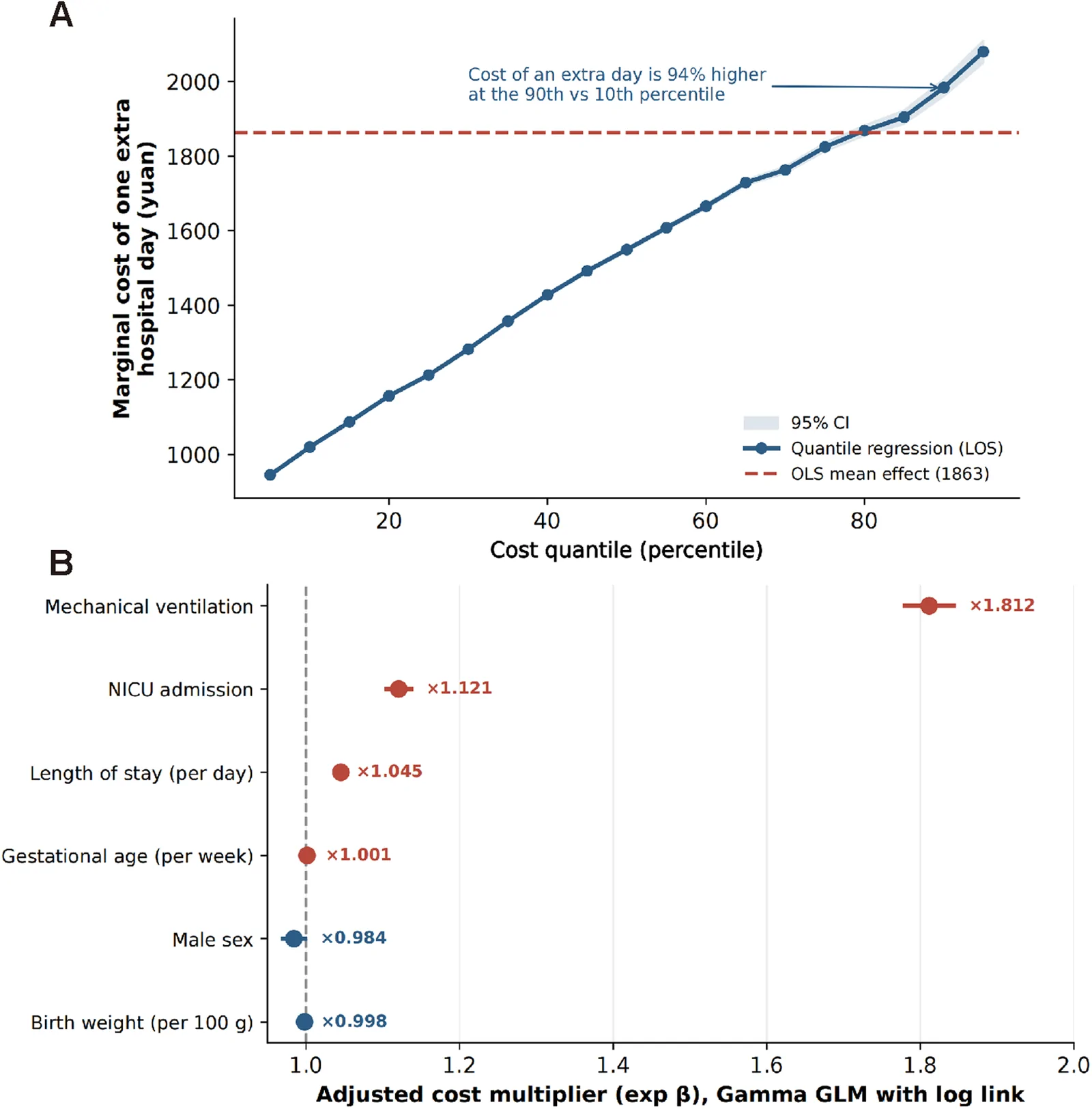
Divergent trends in annual admissions (bars, left axis) and the proportion of extremely preterm infants (born at < 28 weeks of gestation; line, right axis) from 2020 to 2024. Total admissions declined by 34.6%, while the proportion of extremely preterm infants increased 3.5-fold.

The proportion of patients receiving mechanical ventilation increased from 27.1% (808/2,986) in 2020 and 31.5% (865/2,742) in 2021 to 34.9% (824/2,362), 36.1% (711/1,969), and 39.3% (768/1,952) in 2022–2024; overall, the rate was 29.2% (1,673/5,728) in 2020–2021 and 36.7% (2,303/6,283) in 2022–2024, while in-hospital mortality remained below 0.5% in all years. The median total hospitalization cost was 9,841, 10,498, 10,415, 9,588, and 9,775 CNY in 2020 through 2024, respectively, with an overall median of 10,055 CNY. After standardizing to the 2020 GA distribution, the GA-standardized cost indices were 12,811, 15,136, 14,749, 14,020, and 13,734 CNY, respectively, indicating that while the crude median cost remained relatively stable, shifts in GA composition can affect year-to-year cost comparisons (Table 1). Due to changes in NICU ward naming and discharge department recording during the study period, NICU admission rates were not used to directly evaluate annual trends.

### 3.2 Changes in cost structure around the reform index point

ITS analysis showed that the monthly proportion of procedural fees exhibited no statistically significant trend before the January 2022 reform index point (β = 0.33 percentage points/month, 95% CI: −0.11 to 0.78, *P* = 0.141). After the index point, an immediate level increase of 32.30 percentage points was observed (95% CI: 25.81 to 38.79, *P* < 0.001), and the post-index trend decreased by 0.48 percentage points per month relative to the pre-index trend (95% CI: −0.91 to −0.05, *P* = 0.029), yielding an actual post-index trend of −0.15 percentage points per month (95% CI: −0.19 to −0.11, *P* < 0.001). No statistically significant immediate level change or trend change was observed for the proportion of Western medicine fees (all *P* > 0.05). The coefficients of determination for the procedural fee and Western medicine fee models were 0.957 and 0.440, respectively (Table 2; Figure 2). Annual cost composition data showed that the median value of procedural fees increased from 508 CNY to 3,932 CNY, and its proportion rose from approximately 5% to approximately 40% (Figure 3). At the fee-category level, the analyzed procedural fee corresponds to the hospital billing category of general treatment/operation fees, and the increase was concentrated in this category; in the same data, separately itemized nursing fees declined from 11.9% of total billed costs in 2020 and 8.6% in 2021 to 0.9%-1.3% in 2022–2024, suggesting that billing reclassification may have contributed to the apparent procedural-fee jump.

**Table 2:** Segmented regression results from the ITS analysis of monthly cost component proportions.

Parameter	Procedural fee proportion, β (95% CI)	*P* value	Western medicine fee proportion, β (95% CI)	*P* value
Pre-index level, β₀, %	3.12 (−0.26 to 6.50)	0.070	8.03 (7.63 to 8.43)	<0.001
Pre-index monthly trend, β₁, pp/month	0.33 (−0.11 to 0.78)	0.141	−0.03 (−0.07 to 0.01)	0.142
Immediate level change at index, β₂, pp	32.30 (25.81 to 38.79)	<0.001	−0.31 (−1.48 to 0.85)	0.599
Post-index trend change, β₃, pp/month	−0.48 (−0.91 to −0.05)	0.029	0.005 (−0.038 to 0.047)	0.833
Post-index actual trend, β₁+β₃, pp/month	−0.15 (−0.19 to −0.11)	<0.001	−0.03 (−0.06 to 0.01)	0.123
Model (R^2^)	0.957	—	0.440	—

pp, percentage points; ITS, interrupted time series; DRG, diagnosis-related groups; DIP, diagnosis-intervention packet. Monthly discharge settlement was used as the time unit. January 2022 was set as the pre-specified reform index point consistent with the national three-year DRG/DIP payment reform action plan; October through December 2021 were excluded as the transition period. β₀: model-estimated level at the initial study month; β₁: pre-index monthly trend; β₂: immediate level change after the index point; β₃: change in post-index trend relative to the pre-index trend; β₁+β₃: actual post-index monthly trend. Regression coefficients were estimated with Newey-West heteroscedasticity- and autocorrelation-consistent standard errors with a lag order of 6.

**Figure 2:**
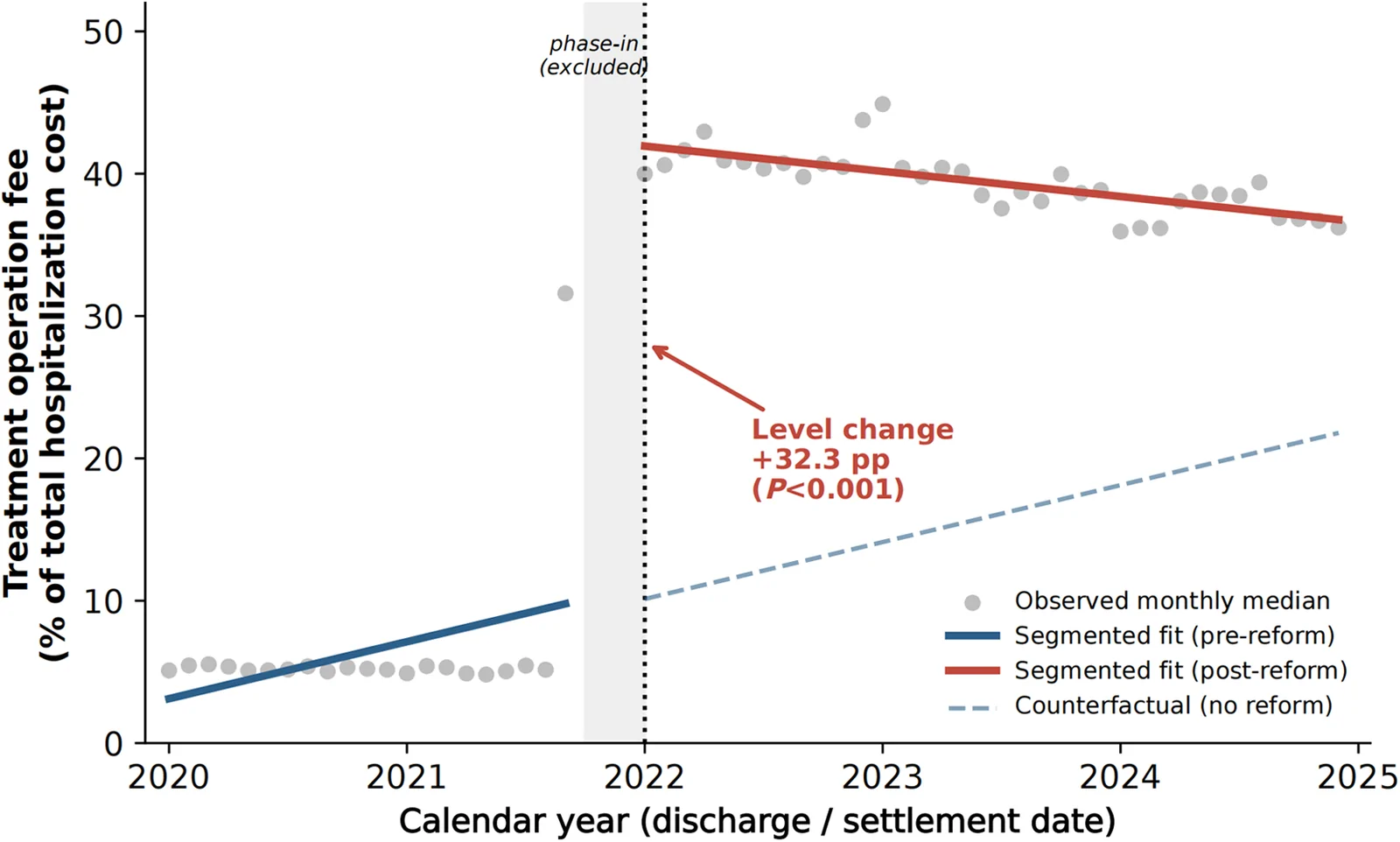
ITS of the monthly proportion of procedural service fees by discharge/settlement month. A stable pre-reform segment is followed by a level change of +32.3 percentage points at January 2022; the dashed line represents the counterfactual projection of the pre-reform trend. October through December 2021 were excluded as the transition period.

**Figure 3:**
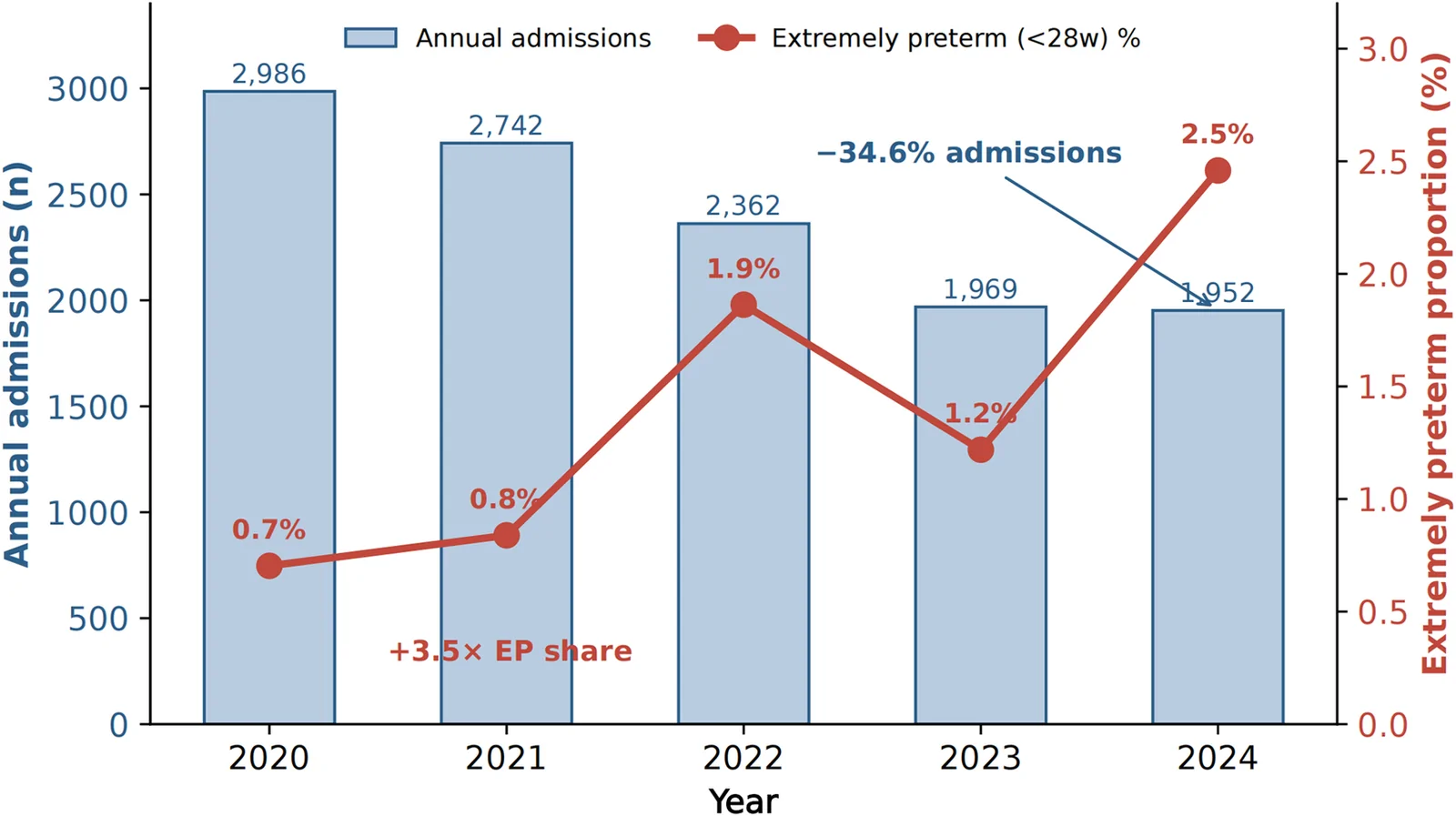
Restructuring of neonatal hospitalization cost composition, 2020 to 2024. After the reform, the proportion of procedural service fees increased from approximately 5% to approximately 40%, while the proportion of Western medicine fees showed no statistically significant concurrent change.

### 3.3 Hospitalization costs and daily cost intensity by gestational age group

Hospitalization costs, LOS, and daily cost intensity all differed significantly across GA groups (all *P* < 0.001; Table 3). Extremely preterm infants had the highest hospitalization costs, with a median of 110,273 CNY (IQR: 25,485 to 161,155 CNY), a median LOS of 62 days (IQR: 8 to 80.5 days), and a daily cost intensity of 1,997 CNY/d (IQR: 1,775 to 3,759 CNY/d). Very preterm infants had a median hospitalization cost of 76,226 CNY (IQR: 44,730 to 102,555 CNY), a median LOS of 44 days (IQR: 27.3 to 56 days), and a daily cost intensity of 1,712 CNY/d (IQR: 1,455 to 1,974 CNY/d). Daily cost intensities were 1,312 CNY/d and 1,160 CNY/d for moderate-to-late preterm and term infants, respectively.

**Table 3:** Daily cost intensity by gestational age group.

GA group	n	Median total cost, CNY [Median (IQR)]	Median LOS, days [Median (IQR)]	Daily cost intensity, CNY/d [Median (IQR)]
Extremely preterm	159	110,273 (25,485–161,155)	62 (8–80.5)	1,997 (1,775–3,759)
Very preterm	570	76,226 (44,730–102,555)	44 (27.3–56)	1,712 (1,455–1,974)
Moderate-to-late preterm	2,994	16,950 (11,950–26,092)	13 (9–19)	1,312 (1,147–1,545)
Term	8,209	8,342 (6,244–11,527)	7 (6–10)	1,160 (987–1,401)

GA, gestational age; LOS, length of stay; IQR, interquartile range; CNY, Chinese Yuan. Daily cost intensity=total hospitalization cost divided by LOS. GA stratification used validated gestational age data (*n* = 11,932); 79 cases with missing GA were excluded from this table.

### 3.4 Cost concentration, disease-specific costs, and length of stay

The Gini coefficient for hospitalization costs was 0.470, 0.489, 0.535, 0.520, and 0.569 in 2020 through 2024, respectively, with an overall coefficient of 0.516, indicating marked heterogeneity in cost distribution and increasing overall concentration over time. In 2024, the top 1%, 5%, 10%, and 20% of patients by cost accounted for 10.9%, 33.7%, 49.2%, and 64.2% of annual total costs, respectively; across all study years, the corresponding proportions were 10.8%, 31.4%, 44.9%, and 59.4% (Table 4; Supplementary Figure S1**)**. Significant differences were observed in hospitalization costs and LOS across primary diagnoses (Supplementary Table S1). NEC and MAS patients had relatively higher hospitalization costs, and NEC patients also had relatively longer LOS.

**Table 4:** Annual cost concentration indices.

Year	Gini coefficient	Top 1%	Top 5%	Top 10%	Top 20%
2020	0.470	9.1%	28.5%	40.7%	55.0%
2021	0.489	12.4%	32.0%	43.7%	56.9%
2022	0.535	10.0%	30.9%	46.1%	61.6%
2023	0.520	11.0%	30.2%	43.7%	59.7%
2024	0.569	10.9%	33.7%	49.2%	64.2%
All years	0.516	10.8%	31.4%	44.9%	59.4%

A Gini coefficient closer to 1 indicates greater concentration of the hospitalization cost distribution. The top 1%, 5%, 10%, and 20% proportions indicate the fraction of total hospitalization costs attributable to patients ranked by descending total cost within the corresponding year or overall period.

### 3.5 Factors associated with hospitalization costs

Univariate analysis indicated that sex, GA, BW, NICU admission, mechanical ventilation, admission season, and LOS were all associated with hospitalization costs (Supplementary Table S2). Lower GA, lower BW, and longer LOS were associated with markedly higher hospitalization costs (all *P* < 0.001). Differences by admission season were also statistically significant (*P* = 0.003), with summer admissions showing the highest median hospitalization costs. OLS regression showed that LOS was the most prominent factor (standardized β = 0.920, *P* < 0.001). Mechanical ventilation and NICU admission were associated with higher hospitalization costs, and increased GA was associated with lower costs; sex was not significantly associated with cost. The model demonstrated high goodness of fit (*R*^2^ = 0.894) with no substantial multicollinearity (VIF < 5 for all variables; Supplementary Table S3).

Quantile regression revealed that the marginal cost per additional day of LOS increased across cost percentiles: 1,022, 1,212, 1,550, 1,823, and 1,983 CNY/d at the 10th, 25th, 50th, 75th, and 90th percentiles, respectively (all *P* < 0.001), with the marginal effect at the 90th percentile being 94.0% higher than at the 10th percentile. Gamma GLM showed that each additional day of LOS was associated with a cost multiplier of 1.045 (approximately 4.5% increase); the cost multipliers for NICU admission and mechanical ventilation were 1.13 and 1.81, respectively. The cost multiplier per 1-g increase in BW was 0.99998 (*P* = 0.003), GA was not significantly associated with cost (*P* = 0.555), and the cost multiplier for male sex was 1.026 (*P* < 0.001) (Table 5; Figure 4).

**Table 5:** Factors associated with hospitalization costs: quantile regression and gamma GLM results.

Analysis method	Quantile/Variable	Effect estimate	*P* value
Quantile regression	P10: LOS, CNY/day	1,022	<0.001
Quantile regression	P25: LOS, CNY/day	1,212	<0.001
Quantile regression	P50: LOS, CNY/day	1,550	<0.001
Quantile regression	P75: LOS, CNY/day	1,823	<0.001
Quantile regression	P90: LOS, CNY/day	1,983	<0.001
Gamma GLM	LOS (per 1-day increase)	1.045 (+4.5%)	<0.001
Gamma GLM	NICU admission	1.13 (+13%)	<0.001
Gamma GLM	Mechanical ventilation	1.81 (+81%)	<0.001
Gamma GLM	BW (per 1-g increase)	0.99998 (−0.002%)	0.003
Gamma GLM	GA (per 1-week increase)	1.001	0.555
Gamma GLM	Male sex	1.026 (+2.6%)	<0.001

LOS, length of stay; NICU, neonatal intensive care unit; BW, birth weight; GA, gestational age; GLM, generalized linear model. Multivariable analyses included 11,929 complete cases. Quantile regression coefficients represent the marginal effect of each additional day of LOS on hospitalization costs at different cost percentiles. Gamma GLM used a log link function; exponentiated regression coefficients (exp β) represent relative cost multipliers.

**Figure 4:**
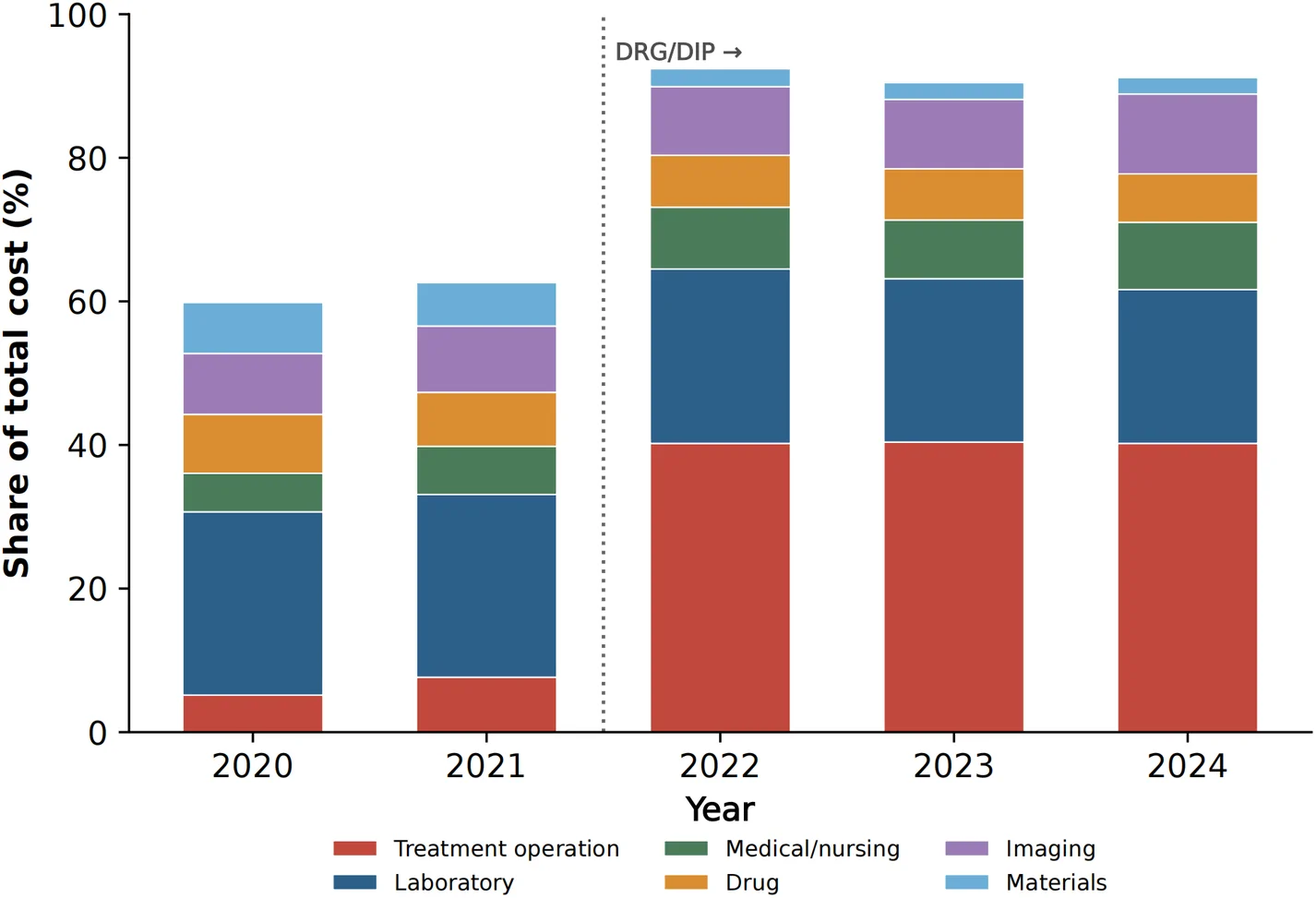
Quantile regression and gamma GLM results. **(A)** Quantile regression: the marginal cost per additional hospital day rises steeply along the cost distribution, far exceeding the OLS mean estimate (dashed line) at the upper tail; the shaded band represents the 95% CI. **(B)** Independent multiplicative cost drivers from Gamma GLM. Points represent adjusted cost multipliers (exp β) with 95% CI; red indicates cost-increasing and blue indicates cost-decreasing factors (relative to reference value of 1.0). Mechanical ventilation (×1.81) and each hospital day (×1.045) were the primary cost amplifiers.

## 4 Discussion

This study analyzed single-center neonatal inpatient data from 2020 to 2024 and yielded four main findings: annual admissions declined markedly while the proportions of extremely preterm infants and mechanically ventilated patients increased; median hospitalization costs remained relatively stable overall, yet cost item composition changed substantially after the January 2022 reform index point; costs became increasingly concentrated among a small subset of resource-intensive patients; and LOS and mechanical ventilation were consistently associated with higher costs across multiple models. These findings collectively suggest that neonatal inpatient case mix, treatment intensity, and cost structure are undergoing notable changes against the backdrop of concurrent demographic shifts and payment system reform.

The decline in admissions is consistent with the nationwide trend of decreasing birth rates in China (1), yet the proportion of extremely preterm infants rose from 0.7% to 2.4% and the mechanical ventilation rate increased year over year. Prior research has shown that advances in perinatal medicine and neonatal intensive care have improved survival for extremely preterm infants while simultaneously increasing the need for intensive monitoring, respiratory support, prolonged hospitalization, and adverse outcome management (1, 10, 11, 17). In the present study, the daily cost intensity of extremely preterm infants was approximately 72% higher than that of term infants, and extremely preterm infants had the longest hospitalizations, underscoring that a decline in admission volume does not necessarily translate into a proportionate reduction in NICU resource demand. NICU bed allocation, staffing, and funding should therefore be guided by GA distribution, disease severity, and treatment intensity rather than annual birth volumes or admission counts alone. Given the small annual number of extremely preterm infants (21, 23, 44, 24, and 47 cases in 2020 through 2024), the decrease in 2023 and increase in 2024 may partly reflect random year-to-year variation. Because the dataset did not include the number of maternal deliveries or referral flows, it cannot determine whether these fluctuations represent changes in population incidence or changes in referral and admission patterns.

This study observed an immediate increase of 32.30 percentage points in the proportion of procedural fees after the January 2022 index point, whereas the proportion of Western medicine fees showed no concurrent step change. Prior evidence indicates that DRG/DIP payment may drive a shift in cost structure away from pharmaceuticals and consumables toward diagnostic, procedural, and medical service items (8, 9); NVBP has primarily affected pharmaceutical expenditure through reductions in drug prices and procurement spending (6, 7, 15, 18). The present findings are consistent with prior evidence of cost composition adjustments, but because a single-arm ITS without a concurrent control group was used, and because the results may reflect changes in billing classification, the COVID-19 pandemic, and other concurrent policies, a causal interpretation is not warranted. A more prudent interpretation is that the advancement of payment reform was temporally concurrent with changes in neonatal inpatient cost composition. This finding suggests that DRG/DIP group weights and reimbursement standards should be dynamically recalibrated in light of post-reform cost structures and recent data. At the fee-category level, the increase was concentrated in the billing category of general treatment/operation fees. Because procedure-level codes were not consistently available across the study period, the data cannot identify specific neonatal procedures responsible for the change. Separately itemized nursing fees declined from 11.9% and 8.6% in 2020 and 2021 to 0.9%–1.3% in 2022–2024, a pattern consistent with a contribution from billing reclassification to the observed shift. Accordingly, the ITS result should be interpreted as a change in billed cost composition rather than evidence of a post-reform change in clinical procedural practice.

Hospitalization costs were highly concentrated, with the top 5% accounting for 31.4% of total costs across all study years and the Gini coefficient rising from 0.470 to 0.569. Prior research has identified low GA, severe complications, and prolonged NICU stays as primary sources of high neonatal costs (10, 19–22). In the present study, NEC had the highest hospitalization costs and longest LOS; NRDS, MAS, and extremely preterm infants also demonstrated high resource consumption, suggesting that cost concentration is primarily driven by disease severity, treatment complexity, and LOS. The complex inflammatory and metabolic characteristics of NEC may further contribute to its substantial resource requirements (23). For a right-skewed, highly concentrated cost distribution, relying solely on mean costs for reimbursement or performance evaluation may systematically underestimate actual resource consumption in high-risk cases. Payment systems should therefore strengthen risk adjustment and consider incorporating GA, mechanical ventilation, severe complications, and prolonged hospitalization as factors in the compensation framework for complex cases.

LOS was the most consistently associated factor with cost in this study. OLS analysis demonstrated the largest standardized effect for LOS, Gamma GLM showed each additional day was associated with approximately 4.5% higher costs, and quantile regression further revealed that the marginal effect increased from 1,022 CNY/d at the 10th percentile to 1,983 CNY/d at the 90th percentile. Prior studies have similarly identified LOS as the primary determinant of costs for preterm infants and NICU patients (12, 21, 22). The quantile regression results further indicate that the impact of LOS is stronger in high-cost patients; thus, cost management strategies should focus particularly on patients with prolonged hospitalization and severe illness rather than applying uniform targets across all patients. Potential approaches to reducing avoidable prolongation of stay include standardizing respiratory support and nutritional management protocols, strengthening infection control, implementing comprehensive multidisciplinary discharge assessments, and promoting family-integrated care; all measures should be implemented without compromising quality of care or patient safety. To distinguish duration of care from per-day resource intensity, daily cost intensity was analyzed alongside cumulative hospitalization cost. Thus, the association between LOS and total cost should be interpreted as reflecting longer resource use and underlying clinical complexity, rather than as evidence that LOS is an independently modifiable causal determinant.

Mechanical ventilation was another important cost-related factor, with Gamma GLM showing that ventilated patients had approximately 1.81 times the costs of non-ventilated patients. Mechanical ventilation reflects both disease severity and the associated requirements for monitoring, medication, procedures, and complication management, consistent with previous neonatal cost studies (20, 21). The cost multiplier for NICU admission was 1.13, which may reflect incomplete capture of care intensity by the NICU variable, the receipt of high-intensity treatment by some patients outside the NICU, and partial overlap between severity effects and LOS or mechanical ventilation. Therefore, mechanical ventilation should be interpreted primarily as a marker of clinical complexity and resource intensity rather than as an independent cost target. Future studies incorporating ventilation duration and respiratory support intensity may further clarify their relationship with neonatal healthcare resource utilization.

These findings have implications for neonatal payment policy and resource allocation. The increasing proportion of high-risk neonates, concentrated healthcare costs, and the important roles of LOS and mechanical ventilation suggest that risk adjustment should be incorporated into neonatal reimbursement systems to better reflect resource requirements.

## 5 Study limitations

Several limitations should be acknowledged. First, this was a single-center retrospective study, and the findings may not be fully generalizable to other healthcare settings. In addition, the observational design allows identification of associations but does not establish causal relationships between clinical factors and hospitalization costs. Although important clinical variables were adjusted for, residual confounding from disease severity, socioeconomic factors, staffing changes, and technological advances cannot be completely excluded. Second, the cost analysis focused on direct hospitalization expenditures and did not capture broader economic consequences, including post-discharge costs and family burden. Furthermore, clinical outcomes were limited to in-hospital outcomes, preventing evaluation of the relationship between resource utilization and long-term health outcomes. Third, the interrupted time series analysis did not include a concurrent control group; therefore, the observed changes in cost composition after the reform index point should be interpreted as temporal associations rather than causal effects. Other concurrent factors, including billing classification changes and healthcare policy changes, may have contributed to the observed trends. Finally, several measurement limitations should be considered. NICU admission status may have been affected by changes in ward classification and recording practices. Mechanical ventilation was analyzed as a binary variable because duration data were unavailable, and detailed procedure-level billing information was insufficient to identify specific procedural changes. Future multicenter studies incorporating standardized severity measures, detailed procedure codes, ventilation duration, and long-term outcomes are warranted.

## 6 Conclusions

From 2020 to 2024, neonatal admissions at this center declined continuously, while the proportions of extremely preterm infants, mechanically ventilated patients, and cost concentration gradually increased, indicating a shift in the demand for neonatal healthcare resources from volume-driven to complexity-driven and high-intensity-treatment-driven. After the payment reform index point, cost composition changed markedly, with an increase in the proportion of procedural fees and no concurrent significant change in the proportion of Western medicine fees. Because this study used a single-arm ITS design, these findings primarily reflect temporal associations and require further validation in multicenter studies with concurrent controls. LOS and mechanical ventilation were important factors associated with increased neonatal hospitalization costs, and high-resource patients contributed disproportionately to total costs. Future neonatal health insurance payment and resource allocation strategies should therefore strengthen risk adjustment by incorporating GA, disease severity, respiratory support requirements, and prolonged hospitalization, thereby improving payment equity and sustaining the capacity to care for high-risk neonates.

## Data Availability

The original contributions presented in the study are included in the article/Supplementary Material, further inquiries can be directed to the corresponding authors.
