# Supplementary material for: Changes in neonatal inpatient resource utilization and cost drivers under payment reform: a five-year retrospective real-world data study
Source: Front Pediatr. 2026 Sep 21;14:1943786. doi: 10.3389/fped.2026.1943786 (PMC13635320; doi:10.3389/fped.2026.1943786)
Supplement: Supplementary file 1 [file Datasheet1.docx]

**Changes in Neonatal Inpatient Resource Utilization and Cost Drivers Under Payment Reform: A Five-Year Retrospective Real-World Data Study**

Jinhui Hu ^1^, Juan Liu ^1^,Jing Qin ^2*^,Zhaojun Pan ^1*^

^1^Neonatal Medical Center, Affiliated Hospital of Yang Zhou University Medical College Huai’an maternal and child health care center, Huai’an, China

^2^Department of Finance, Affiliated Hospital of Yang Zhou University Medical College Huai’an maternal and child health care center, Huai’an, China

^*^These authors contributed equally to this work

Correspondence:

Jing Qin, Tel, +86-13905235281; Email, qinjing1124@126.com

Zhaojun Pan, Tel, +86-13805233269; Email, [13805233269@yzu.edu.cn](mailto:13805233269@yzu.edu.cn)

**Supplementary Methods**

1. **Derived Indicators and Statistical Models**

**(1) Gestational age (GA)-standardized cost index and daily cost intensity.** Using the GA subgroup distribution in 2020 as the reference weights, cost metrics for each annual GA subgroup were weighted to derive the GA-standardized cost index, which reflects the cost level in each year under a standardized GA distribution. This index controls only for differences in GA composition and is not equivalent to a fully case-mix-adjusted estimate or the overall median cost. Daily cost intensity is defined as total hospitalization cost divided by length of stay (LOS) and is used to compare per-unit-time resource consumption across GA groups (14).

**(2) Interrupted time series (ITS) analysis.** Based on the national three-year diagnosis-related groups (DRG)/diagnosis-intervention packet (DIP) payment reform action plan and the local progress of reform implementation, January 2022 was set as the index point, and October through December 2021 were excluded as the transition period. A segmented regression model with the monthly proportion of procedural fees as the outcome was constructed to assess level and trend changes before and after the index point, using Newey-West robust standard errors to account for autocorrelation and heteroscedasticity. The same model was applied to the monthly proportion of Western medicine fees as a comparative analysis. Because no concurrent control group was available, results are interpreted only as temporal associations around this index point (15).

**(3) Cost concentration.** Lorenz curves and the Gini coefficient were used to characterize the distribution of hospitalization costs. The proportions of total costs attributable to the top 1%, 5%, 10%, and 20% of patients (ranked by descending cost) were also calculated. A Gini coefficient closer to 1 indicates greater concentration of the cost distribution (16).

**(4) Regression models.** Gamma generalized linear model (GLM) with a log link function and total hospitalization cost as the dependent variable served as the primary multivariable model; exponentiated regression coefficients (exp β) represent cost multiplier effects. Quantile regression was used to assess the marginal effect of LOS at different cost percentiles. Ordinary least squares (OLS) linear regression was included as a supplementary analysis. Candidate variables included sex, GA, birth weight (BW), neonatal intensive care unit (NICU) admission, mechanical ventilation, and LOS.

**2. Disease-Specific Hospitalization Costs and Length of Stay**

Hospitalization costs and LOS differed markedly across primary diagnoses (**Supplementary Table 1**). Necrotizing enterocolitis (NEC) had the highest median hospitalization cost at 74,662 CNY (interquartile range, IQR: 48,025 to 137,884 CNY) and the longest median LOS at 35 days (IQR: 26.5 to 68 days). Median hospitalization costs for meconium aspiration syndrome (MAS) and neonatal respiratory distress syndrome (NRDS) were 57,677 CNY (IQR: 38,232 to 86,616 CNY) and 51,498 CNY (IQR: 30,895 to 89,074 CNY), respectively. Hypoxic-ischemic encephalopathy (HIE) and central nervous system (CNS) infection had median costs of 32,794 CNY and 24,064 CNY, respectively.

**Supplementary Table 1. Hospitalization costs and length of stay by major diagnosis**

| **Primary diagnosis** | **Median cost, CNY [Median (IQR)]** | **Cost rank** | **Median LOS, days [Median (IQR)]** | **LOS rank** |
| --- | --- | --- | --- | --- |
| Necrotizing enterocolitis (NEC) | 74,662 (48,025–137,884) | 1 | 35 (26.5–68) | 1 |
| Meconium aspiration syndrome (MAS) | 57,677 (38,232–86,616) | 2 | 15 (10–18) | 5 |
| Neonatal respiratory distress syndrome (NRDS) | 51,498 (30,895–89,074) | 3 | 29 (17–49) | 2 |
| Hypoxic-ischemic encephalopathy (HIE) | 32,794 (26,297–43,254) | 4 | 15.5 (12.3–20.8) | 4 |
| CNS infection | 24,064 (16,398–37,922) | 5 | 21 (17–29) | 3 |

*NEC: necrotizing enterocolitis; MAS: meconium aspiration syndrome; NRDS: neonatal respiratory distress syndrome; HIE: hypoxic-ischemic encephalopathy; CNS: central nervous system; LOS: length of stay; IQR: interquartile range; CNY: Chinese Yuan. Data are median (IQR). Diagnoses are ranked by median hospitalization cost.*

**3. Univariate Analysis of Factors Associated with Hospitalization Costs**

All 12,011 validated patients were included in univariate analyses; effective sample sizes varied slightly across variables due to missing values. Univariate analyses showed that sex, GA, BW, NICU admission, mechanical ventilation, admission season, and LOS were all associated with hospitalization costs (**Supplementary Table 2**). Infants with GA < 32 weeks had a median cost of 78,747 CNY, compared with 16,950 CNY for infants at 32 to 36+6 weeks and 8,343 CNY for term infants (*P* < 0.001). Infants with BW < 1,500 g had a median cost of 86,875 CNY (*P* < 0.001). Median costs were 8,580 CNY, 25,131 CNY, and 80,180 CNY for patients with LOS of 14 days or fewer, 15 to 28 days, and more than 28 days, respectively (*P* < 0.001).

**Supplementary Table 2. Univariate analysis of factors associated with hospitalization costs**

| **Factor** | **Category** | **n** | **Median cost, CNY** | **P value** |
| --- | --- | --- | --- | --- |
| Sex | Male / Female | 6,804 / 5,207 | 10,473 / 9,675 | <0.001 |
| GA | <32 / 32–36+6 / ≥37 wk | 729 / 2,994 / 8,209 | 78,747 / 16,950 / 8,343 | <0.001 |
| BW | <1,500 / 1,500–2,499 / ≥2,500 g | 578 / 1,990 / 9,436 | 86,875 / 21,490 / 8,822 | <0.001 |
| NICU admission | Yes / No | 6,048 / 5,963 | 11,825 / 8,563 | <0.001 |
| Mechanical ventilation | Yes / No | 3,976 / 8,035 | 21,190 / 7,896 | <0.001 |
| Admission season | Spring / Summer / Autumn / Winter | 2,984 / 2,924 / 2,804 / 3,299 | 10,002 / 10,497 / 10,015 / 9,798 | 0.003 |
| LOS | ≤14 / 15–28 / >28 days | 9,529 / 1,612 / 870 | 8,580 / 25,131 / 80,180 | <0.001 |

*GA: gestational age; BW: birth weight; NICU: neonatal intensive care unit; LOS: length of stay; CNY: Chinese Yuan. Comparisons were performed using the Kruskal-Wallis H test for variables with more than two categories and the Mann-Whitney U test for two-category variables. 79 cases had missing GA; 7 cases had missing BW.*

**4. Multivariable Linear (OLS) Regression Analysis of Hospitalization Costs**

The OLS supplementary analysis included 11,929 complete cases. Each additional day of LOS was associated with an average increase of 1,864 CNY (95% CI: 1,848 to 1,879 CNY, *P* < 0.001), which had the largest standardized regression coefficient (β = 0.920). Mechanical ventilation was associated with an increase of 2,898 CNY (95% CI: 2,481 to 3,316 CNY, *P* < 0.001); NICU admission was associated with an increase of 2,384 CNY (95% CI: 2,049 to 2,719 CNY, *P* < 0.001); each additional week of GA was associated with a decrease of 136 CNY (*P* = 0.009); and male sex was not significantly associated with cost (*P* = 0.198). The model *R*² was 0.894, and all VIF values were below 5 (**Supplementary Table 3**).

**Supplementary Table 3. Multivariable linear (OLS) regression of hospitalization costs**

| **Variable** | **B (CNY)** | **Standardized β** | **95% CI** | ***P* value** |
| --- | --- | --- | --- | --- |
| LOS (days) | 1,864 | 0.920 | 1,848 to 1,879 | <0.001 |
| Mechanical ventilation | 2,898 | 0.049 | 2,481 to 3,316 | <0.001 |
| BW (g) | 0.83 | 0.024 | 0.49 to 1.18 | <0.001 |
| NICU admission | 2,384 | 0.043 | 2,049 to 2,719 | <0.001 |
| GA (weeks) | −136 | −0.015 | −238 to −35 | 0.009 |
| Male sex | 217 | 0.004 | −113 to 547 | 0.198 |

*OLS: ordinary least squares; LOS: length of stay; BW: birth weight; NICU: neonatal intensive care unit; GA: gestational age; VIF: variance inflation factor; CNY: Chinese Yuan. R² = 0.894 (explaining 89.4% of the variance); analysis sample n = 11,929; VIF < 5 for all predictors. B = unstandardized regression coefficient.*

**Supplementary Figure 1. A.** Lorenz curve of neonatal hospitalization costs (overall Gini coefficient = 0.516); the most expensive 5% of patients accounted for 31.4% of total expenditure. **B.** Annual Gini coefficients increased from 0.470 in 2020 to 0.569 in 2024.
